# Parallel evolution of opsin visual pigments in hawkmoths by tuning of spectral sensitivities during transition from a nocturnal to a diurnal ecology

**DOI:** 10.1242/jeb.244541

**Published:** 2022-12-12

**Authors:** Tokiho Akiyama, Hironobu Uchiyama, Shunsuke Yajima, Kentaro Arikawa, Yohey Terai

**Affiliations:** ^1^ Department of Evolutionary Studies of Biosystems, SOKENDAI (The Graduate University for Advanced Studies), Shonan Village, Hayama, Kanagawa 240-0193, Japan; ^2^ NODAI Genome Research Center, Tokyo University of Agriculture, 1-1-1 Sakuragaoka, Setagaya, Tokyo 156-8502, Japan; ^3^ Department of Bioscience, Tokyo University of Agriculture, 1-1-1 Sakuragaoka, Setagaya, Tokyo 156-8502, Japan

**Keywords:** Parallel evolution, Visual adaptation, Opsin, Spectral sensitivity, Diurnal–nocturnal transition, Hawkmoth

## Abstract

Light environments differ dramatically between day and night. The transition between diurnal and nocturnal visual ecology has happened repeatedly throughout evolution in many species. However, the molecular mechanism underlying the evolution of vision in recent diurnal–nocturnal transition is poorly understood. Here, we focus on hawkmoths (Lepidoptera: Sphingidae) to address this question by investigating five nocturnal and five diurnal species. We performed RNA-sequencing analysis and identified opsin genes corresponding to the ultraviolet (UV), short-wavelength (SW) and long-wavelength (LW)-absorbing visual pigments. We found no significant differences in the expression patterns of opsin genes between the nocturnal and diurnal species. We then constructed the phylogenetic trees of hawkmoth species and opsins. The diurnal lineages had emerged at least three times from the nocturnal ancestors. The evolutionary rates of amino acid substitutions in the three opsins differed between the nocturnal and diurnal species. We found an excess number of parallel amino acid substitutions in the opsins in three independent diurnal lineages. The numbers were significantly more than those inferred from neutral evolution, suggesting that positive selection acted on these parallel substitutions. Moreover, we predicted the visual pigment absorption spectra based on electrophysiologically determined spectral sensitivity in two nocturnal and two diurnal species belonging to different clades. In the diurnal species, the LW pigments shift 10 nm towards shorter wavelengths, and the SW pigments shift 10 nm in the opposite direction. Taken together, our results suggest that parallel evolution of opsins may have enhanced the colour discrimination properties of diurnal hawkmoths in ambient light.

## Teaser

**Highlighted Article:** Nocturnal to diurnal transitions in hawkmoths are accompanied by reduced separation between short- and long-wavelength sensitivity peaks which may be caused by parallel amino acid substitutions in visual opsins.

## INTRODUCTION

Adaptive evolution of the visual system to various light environments is a profound factor in the diversification of organisms, which sometimes drives speciation (Walls, 1942; Terai and Okada, 2011). The transition between diurnal and nocturnal ecology has happened repeatedly throughout evolutionary history (Maor et al., 2017; Kawahara et al., 2018). The light environments differ between clear and cloudy skies during the day, and between full moon and starlight at night. However, the most drastic difference is the variation in the light environment between day and night (Johnsen et al., 2006). Since both the intensity and wavelength composition of sky light vary between day and night in terrestrial habitats, the differences in ambient light are considered to have affected the spectral sensitivity of retinal photoreceptors (Cronin et al., 2014).

The visual response starts when the visual pigment molecules in the photoreceptor cells absorb light (Land and Nilsson, 2012). The visual pigment consists of an opsin protein, a GTP binding protein-coupled receptor (GPCR), with an 11-*cis* retinal attached as the chromophore (Terakita, 2005). The amino acid sequence of opsin and the type of chromophore determine the visual pigment's absorption spectrum (Yokoyama, 2000).

The repertoire of visual opsin genes has often changed with evolutionary transitions of daily activity patterns (Kawamura, 2011; Liu et al., 2018). For example, the ‘nocturnal bottleneck’ hypothesis during early mammalian evolution (Walls, 1942; Gerkema et al., 2013) suggests that the common ancestor of vertebrates was diurnal and had colour vision based on four cone opsins. Then, the ancestors of placental mammals lost two of the four cone opsins and became nocturnal for over 100 million years during the Mesozoic era. After extinction of the dinosaurs, mammals expanded to be diurnal (Maor et al., 2017). One of the remaining opsin genes then duplicated independently in the common ancestor of catarrhine primates (Old World monkeys, apes and humans) and New World howler monkeys (Surridge et al., 2003).

An analogous example is known in the evolution of lepidopteran insects, moths and butterflies. Roughly 75–85% of lepidopteran species are nocturnal, but their common ancestor appears to have had a diurnal ecology (Kawahara et al., 2018). In the order Lepidoptera, the common ancestor of Heteroneura became nocturnal around 209.7 million years ago (MYA). An ancestral lineage of butterflies (Papilionoidea) shifted back to a diurnal lifestyle around 98.3 MYA (Kawahara et al., 2019). The common ancestor of butterflies presumably had three opsins, which are duplicated or lost in some species (Briscoe, 2008; Feuda et al., 2016; Sondhi et al., 2021).

However, we know little about the effect of recent diurnal–nocturnal transition on opsin gene evolution. How does such a transition with the drastic change in light environment affect vision? Do the opsin genes evolve rapidly? To approach these questions, we need to analyse the effects of diurnal–nocturnal transitions on opsins in a set of closely related species.

Hawkmoths (Lepidoptera: Sphingidae) provide an ideal opportunity to address these questions. The hawkmoth family appeared around 42.8 MYA and the subtribe Choerocampina appeared around 12.2 MYA (Kawahara et al., 2019); thus, the subfamily Macroglossinae containing both nocturnal and diurnal species that are phylogenetically close appeared between these two estimated ages (*Sphingidae of the Eastern Palaearctic*; http://tpittaway.tripod.com/china/china.htm; accessed 8 March 2022) (Kishida, 2011). Extensive phylogenetic analysis has indicated that the ancestral hawkmoth was nocturnal (Kawahara et al., 2018; 2019). In the subfamily Macroglossinae, some species became diurnal within a short evolutionary time period (Kawahara et al., 2009; 2018; Chen et al., 2021).

The hawkmoths' visual system has been studied extensively. The diurnal Hummingbird hawkmoth *Macroglossum stellatarum*, the nocturnal Elephant hawkmoth *Deilephila elpenor* and the nocturnal Tobacco hawkmoth *Manduca sexta*, all use colour vision for floral foraging (Kelber, 1997; Kelber et al., 2002; Goyret et al., 2008). In both diurnal and nocturnal species, the compound eyes are the refracting superposition type adaptive to scotopic vision (Nilsson, 1989; Warrant et al., 1999). Such enigmatic phenomena in hawkmoth vision, i.e. colour vision in the nocturnal species and scotopic vision-type compound eyes in the diurnal species, suggest that they have experienced diurnal–nocturnal transitions rather recently.

Previous studies on *M. stellatarum* and *M. sexta* have revealed that both possess a set of three opsin genes: ultraviolet-sensitive (UV), short wavelength-sensitive (SW) and long wavelength-sensitive (LW) (Chase et al., 1997; Xu et al., 2013; Kanost et al., 2016). Their opsins presumably carry key information on what happened to the evolution of visual perception at the molecular level during diurnal–nocturnal transition events, which occurred recently relative to such events in primates and butterflies.

Therefore, we examine the hawkmoth visual opsins to shed light on how their vision has evolved through the ecological transition from nocturnal to diurnal activities among closely related species. We identified the opsin genes, their expression levels and possible absorption spectra in multiple hawkmoth species. We show that hawkmoth species most likely have adapted their visual function in parallel during changes in their ecology from a nocturnal to a diurnal lifestyle.

## MATERIALS AND METHODS

### Samples

For molecular biological experiments, we used 10 hawkmoth species (Lepidoptera: Sphingidae): five nocturnal and five diurnal. We caught adult individuals of the following species in almost the same geographic location (especially for the diurnal species): nocturnal: *Marumba gaschkewitschii* and *Ambulyx ochracea* in subfamily Smerinthinae; *Ampelophaga rubiginosa* and *Theretra japonica* in subfamily Macroglossinae; diurnal: *Cephonodes hylas*, *Hemaris affinis*, *Neogurelca himachala*, *Macroglossum pyrrhosticta* and *Macroglossum bombylans* in subfamily Macroglossinae. Adult individuals were kept at 25°C under a 14 h light:10 h dark cycle for 1–5 days before use. We also collected the larvae of nocturnal *Daphnis nerii* in the subfamily Macroglossinae and then reared them on the fresh leaves of *Catharanthus roseus* and kept the pupae at 28°C under a 14 h light:10 h dark cycle for 16–22 days. We used the adults within 2 days of emergence. We sampled two or three individuals for all 10 species for RNA-sequencing (RNA-seq) analysis (Table S1).

We performed electrophysiological and histological experiments in two nocturnal and two diurnal species: *M. gaschkewitschii* (*n*=20: 18 males and 2 females for electrophysiology; *n*=1 male for histology), *C. hylas* (*n*=18: 16 males and 2 females for electrophysiology; *n*=1 male for histology), *M. pyrrhosticta* (*n*=18: 9 males and 9 females for electrophysiology; *n*=1 male for histology) and *T. japonica* (*n*=19: 14 males and 5 females for electrophysiology; *n*=1 male for histology). We selected these species because they belong to different clades of the species tree. The numbers of samples were determined to ensure stable results of analyses. Larvae or adults of these species were caught in the same area as those used for molecular experiments. The larvae were reared on the fresh leaves of their host plants at 25°C under a 14 h light:10 h dark cycle for 3–44 days, and the pupae were stored at 10°C for 2–12 months or left at 25°C for 17–24 days and allowed to emerge at 25°C. The adults were kept for 1–40 days at 10°C under a 14 h light:10 h dark cycle and occasionally fed with 15% sucrose solution, except for *M. gaschkewitschii*, which does not take food, until use.

The species names and the classification were based on the literature (Sphingidae Taxonomic Inventory, http://sphingidae.myspecies.info/; accessed 8 March 2022) (Kitching and Cadiou, 2000; Kishida, 2011). Sex was identified by genital morphology. Their ecology, such as nocturnal and diurnal, was based on *Sphingidae of the Eastern Palaearctic* (http://tpittaway.tripod.com/china/china.htm; accessed 8 March 2022), Esaki et al. (1971), Kishida (2011) and our field observations (Table S1).

This research was approved by the animal protocols and procedures committee at SOKENDAI (The Graduate University for Advanced Studies).

### RNA extraction and sequencing

The eyes and brain tissues were dissected under a dissection microscope and preserved in RNA*later* solution (Ambion, Austin, TX, USA). The dissection was done between 12:00 h and 13:00 h. Total RNAs were extracted from the tissues using the TRIzol reagent (Invitrogen, Carlsbad, CA, USA) following the manufacturer's instructions. From 2 µg of the total RNAs, we purified mRNA by using the NEBNext Poly(A) mRNA Magnetic Isolation Module (New England BioLabs, Ipswich, MA, USA), and constructed the RNA-seq libraries with approximately 400 bp length on average using the NEBNext Ultra RNA Library Prep Kit for Illumina (New England BioLabs) following the manufacturer's instructions. Paired-end reads of 100 bp were determined using an Illumina Hiseq2500 platform (Illumina Inc, San Diego, CA, USA). Short cDNA sequences (2.0–4.0 Gb for each library) were deposited into the DDBJ Sequence Read Archive (DRA) database (accession number: DRA010599) (Table S1).

### Reconstruction of hawkmoth species tree

The RNA-seq data from the adult heads of *M. sexta* (accession no.: SRX702703) and *Helicoverpa armigera* (SRX3595764) were downloaded from the European Nucleotide Archive (ENA) database. *H. armigera* was used as an outgroup. The RNA-seq short reads from 12 species (10 from the present study and 2 from the database) were assembled, aligned and concatenated using the SISRS v1.6 software (Schwartz et al., 2015). A phylogenetic tree was inferred by using the Maximum Likelihood (ML) method based on the General Time Reversible (GTR) model of sequence evolution with discrete gamma-distributed rates among sites (Nei and Kumar, 2000), which was selected as the best fit of the nucleotide substitution model in the MEGA v7.0 software (Kumar et al., 2016). All positions with gaps and ambiguous data were removed from the alignment, resulting in 17,305 positions in the final dataset. The reliability of the tree topology was evaluated using 1000 bootstrap replicates (Felsenstein, 1985). The phylogenetic tree was visualised using FigTree v.1.4.3 (http://tree.bio.ed.ac.uk/software/figtree/).

### Identification of opsin cDNA sequences

After removal of adaptor sequences and low-quality reads (quality score<0.05), we conducted *de novo* assemblies of the short cDNA sequences using the CLC genomics workbench 11.0.1 (https://www.qiagenbioinformatics.com/) for each individual. The quality of the transcriptome assemblies was evaluated by using the Benchmarking Universal Single-Copy Orthologue (BUSCO) v5.4.3 tool with the arthropoda OrthoDB v10 (https://www.orthodb.org/) dataset of 1013 single-copy orthologues (Manni et al., 2021). We isolated the homologous partial sequences from the assembled contigs using a BLASTN search (Altschul et al., 1990) with three visual opsin gene sequences from *M. sexta, Manop1* (LW; GenBank accession no.: L78080.1), *Manop2* (UV; L78081.1), and *Manop3* (SW; AD001674.1), as queries. Using the 50 bases of 5′- and 3′-ends of the homologous partial sequences as the queries, we isolated homologous sequences from the RNA-seq reads by BLASTN search (Altschul et al., 1990) and connected the sequences to both ends of the homologous partial sequences. We repeated this process and then predicted the entire coding region with 5′- and 3′-untranslated regions (UTRs) of opsin cDNAs from each species.

We verified the assembled opsin sequences by polymerase chain reaction (PCR). The total RNAs used for RNA sequencing were purified via chloroform extraction and isopropanol precipitation. First-strand cDNAs were synthesised from 1 µg total RNA using the PrimeScript II 1st Strand Synthesis Kit (TaKaRa Bio Inc., Shiga, Japan). We designed PCR primers on the predicted 5′- and 3′-UTR sequences to amplify the opsin cDNA sequences (Table S2). The cDNAs were used as templates for PCR in a 30 μl solution containing dNTP at 0.25 mmol l^−1^, 0.33 μmol l^−1^ each primer, 0.5 U Ex Taq HS polymerase (TaKaRa Bio Inc.) and the reaction buffer attached to the polymerase. Reactions for all the primer sets were carried out in a Mastercycler (Eppendorf, Hamburg, Germany) using the same PCR conditions: an initial denaturing step at 93°C for 3 min, 30 cycles of denaturation at 93°C for 1 min, annealing at 55°C for 1 min, extension at 72°C for 2 min, and a final extension step at 72°C for 1 min. The PCR products were purified and the sequences were determined with the primers for PCR or sequencing (Table S2) using the BigDye Terminator v3.1 Cycle Sequencing Kit (Applied Biosystems, Foster City, CA, USA) and an Applied Biosystems Automated 3130xl DNA Sequencer (Applied Biosystems, Waltham, MA, USA). The sequences were verified by reading both strands. The opsin cDNA sequences determined in this study were deposited in the international nucleotide sequence database DDBJ/EMBL/GenBank (accession numbers: LC573512–LC573541).

### Analysis of opsin gene expression

For quantification of opsin gene expression, the RNA-seq reads were mapped to the three determined opsin cDNA sequences using CLC genomics workbench 11.0.1 for each individual. The reads per kilobase of exon model per million mapped reads (RPKM) were calculated to normalise the mRNA expression values (Mortazavi et al., 2008). We calculated the mean RPKM value with standard error (s.e.) from two or three individuals for each opsin gene and compared the relative ratios between the three opsin genes in each species. The comparison of the RPKM values between nocturnal and diurnal species for each opsin gene was also assessed by the Wilcoxon rank-sum test using the package ‘exactRankTests’ (https://CRAN.R-project.org/package=exactRankTests) in R v.3.3.3 (https://www.r-project.org/). *P*<0.05 was considered a statistically significant difference.

### Construction of opsin phylogenetic trees

In addition to the opsin sequences determined in the present study, we used three opsin sequences from *M. sexta*, *M. stellatarum* (GenBank accession nos.: UV, KF539456.1; SW, KF539426.1; LW, KF539444.1) and *H. armigera* (UV, KF539454.1; SW, KF539433.1; LW, KF539442.1) for construction of opsin gene trees. We aligned the sequences using CLUSTAL W (Thompson et al., 1994) in MEGA v7.0 software (Kumar et al., 2016), followed by visual inspection. Gene trees of orthologous opsin genes were constructed by the ML method. The Jones–Taylor–Thornton (JTT)+G model for UV and SW opsins (Jones et al., 1992) and the Le and Gascuel, 2008 (LG)+G model (Le and Gascuel, 2008) for LW opsin were selected as the best fit model of the amino acid substitution for the ML tree construction using MEGA. All positions containing gaps were removed from the alignment. The final data set contained 376 positions for UV opsin, 380 positions for SW opsin, and 377 positions for LW opsin. The statistical reliability of the tree branches was evaluated using 1000 bootstrap replicates (Felsenstein, 1985). We used FigTree v.1.4.3 for the visualisation of the phylogenetic trees. We tested the ML gene tree topologies by comparing them to the topology of the hawkmoth species tree that we hypothesised to be the true gene tree topology, using the approximately unbiased (AU) test (Shimodaira, 2002) with 10,000 bootstrap replicates by the resampling estimated log-likelihood (RELL) method (Kishino et al., 1990) in the software IQ-TREE v.1.6.12 (Nguyen et al., 2015). The ML gene tree was considered to be significantly supported at the 95% level (*P*≧0.95).

To evaluate the positions of the amino acid replacements in the structure of opsin proteins, the seven transmembrane regions of each of the three opsin proteins for *M. sexta* as a representative of hawkmoths were predicted by the TMHMM-2.0 server (Sonnhammer et al., 1998; Krogh et al., 2001). By using MEGA, the amino acid sequences of three hawkmoth opsins were aligned with the rhodopsin-1 (Rh1, Kumopsin1; PDB accession no.: 6I9K_A) of a jumping spider (*Hasarius adansoni*) whose crystal structure has been published (Varma et al., 2019). The minimum distance between the retinal and each amino acid residue was examined based on the three-dimensional (3D) structure of jumping spider Rh1 using the iCn3D 2.10 viewer (Wang et al., 2020) in the Molecular Modeling Database (MMDB) (Madej et al., 2014). For visualisation of the 3D structure of the protein, we executed the PyMOL Molecular Graphics System v.2.3.0 (https://pymol.org). Throughout the paper, the amino acid residue numbers were based on those of each opsin in *M. sexta*.

### Comparison of dN/dS (ω) of opsin genes

To elucidate whether the evolutionary rates of amino acid substitution changed between the nocturnal and diurnal branches, we performed ML calculations to estimate synonymous (dS) and non-synonymous (dN) substitution rates and their ratio (dN/dS=ω), for the three opsin genes in the 12 hawkmoth species. We considered two models: the simplest model assuming the same ω value across the tree (one-ratio model), and the model allowing variable ω values between nocturnal and diurnal branches (two-ratio model). The simpler one-ratio model was nested in the two-ratio model. The likelihoods of the former and latter models were compared to test whether the ratio for diurnal branches was the same as the ratio for nocturnal ones. Log-likelihood (lnL) and ω at branches were calculated by the branch model using the program codeml in the PAML 4.8a package (Yang, 1997; 2007) with PAMLX 1.3.1 (Xu and Yang, 2013). The fit of branch models was assessed using the likelihood ratio test (LRT) by χ^2^ test to the 2× (log-likelihood difference) (2 ΔlnL), with the one-ratio model rejected where *P*<0.05. We used the alignments of the opsin cDNA nucleotide sequences from which indels were removed. The analysis was performed based on the topology of the hawkmoth species tree presented in this study. *M. stellatarum*, which was not included in the species tree, was combined to form a cluster with *M. bombylans* because *M. stellatarum* and *M. bombylans* were monophyletic in the three opsin phylogenetic trees. The branches forming the monophyletic groups of diurnal species were defined as diurnal branches.

### Statistical test for parallel evolution of opsin amino acid sequences

**Fig. 1.**
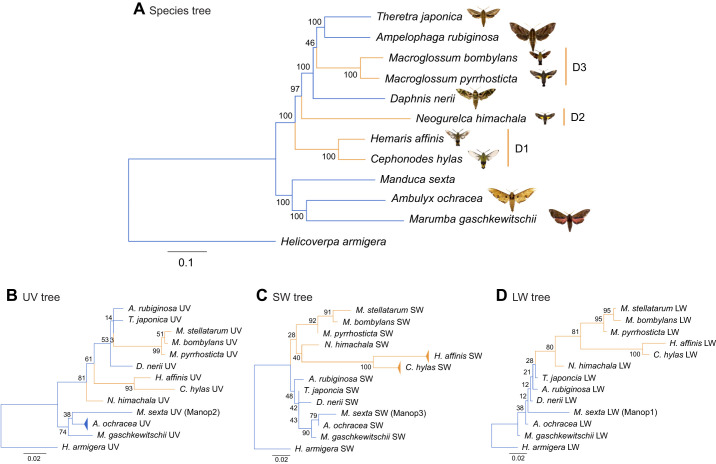
**Phylogenetic trees for hawkmoth species and opsins.** (A) A maximum likelihood (ML) tree for hawkmoth species (Sphingidae) constructed from assembled, aligned and concatenated RNA-seq reads. *H. armigera* was used as an outgroup species. Scale bar indicates 0.1 nucleotide substitutions per site. D1–D3 are the diurnal clades. Photographs of the hawkmoths collected in this study are at the same magnification. (B) UV, (C) SW, and (D) LW opsin gene trees constructed by the ML method. Each orthologous opsin of *H. armigera* was used as an outgroup. The polymorphic branches are collapsed. Scale bar indicates 0.02 amino acid substitutions per site. The bootstrap values are shown at the nodes. The indigo branches are nocturnal, and the orange branches are diurnal.

First, we searched for the opsin amino acid substitutions that occurred in parallel in the three different diurnal clades (D1–D3 in Fig. 1A). For the three opsins, we reconstructed the ancestral states at the nodes by the ML method (Nei and Kumar, 2000) under the JTT matrix-based model with a gamma distribution for rates among sites (Jones et al., 1992), which was chosen as the best fit model using MEGA v7.0 software (Kumar et al., 2016). The analysis was performed based on the topology of the hawkmoth species tree presented in this study. We identified the parallel-substitution sites between the three independent diurnal lineages according to the definition of Zhang and Kumar (1997).

Next, we analysed whether the parallel substitutions of amino acids occurred by chance or not. We used the hawkmoth opsin amino acid sequences and the species tree topology, and obtained the assigned notation of the nodes using the program ancestor in the ANCESTOR package (Zhang and Nei, 1997). Two focused diurnal lineages in the different clades were selected for the statistical tests of parallel evolution of amino acid sequences. When the focused clade contained more than one diurnal species, one sequence was used for the analysis. We counted the total number of parallel-substitution sites on the lineage from the interior nodes, where the common ancestor of the diurnal species diverged from the nocturnal species, to the exterior nodes of the tree for the lineage pair. We performed a Poisson test to verify whether the observed number of parallel substitutions was significantly larger than the expected number because of random substitution under the JTT-f_gene_ amino acid substitution model (Cao et al., 1994) using converg2 in the CAPE package (Zhang and Kumar, 1997). At a significance level of *P*=0.05, we rejected the null hypothesis that the observed parallel substitutions were simply attributed to random chance.

### Electrophysiology of compound eye spectral sensitivity

To examine the absorption spectra of visual pigments, we determined compound eye spectral sensitivities in the four hawkmoth species by recording electroretinograms (ERGs). Under dim red light, we fixed a moth onto a plastic stage with beeswax. We inserted a chloridised silver wire into the stump of an antenna to serve as the reference electrode. After making a tiny crack on the cornea with a razor blade, we mounted the sample in a Faraday cage covered with a blackout curtain. We then inserted the tip of a glass microelectrode filled with physiological saline into the eye through the crack using a micromanipulator. An end-of-quartz optical fibre (Φ3 mm) for light stimulation was placed close to the eye, and its position was fine-tuned to maximise the responses to dim flashes. Before recording the ERG, the sample was left in the dark for at least 2 h in the nocturnal species and at least 30 min in the diurnal species so that the eye was dark-adapted.

The light was provided by a 500 W xenon arc lamp (UXL-500D-O, Ushio Inc., Tokyo, Japan) through a series of 23 narrow-band interference filters ranging from 300 to 740 nm at 20 nm intervals (full width at half maximum, FWHM=10–14 nm, Asahi Spectra, Tokyo, Japan). Light intensity was controlled by a set of neutral density (ND) filters. The photon flux of each monochromatic light was measured using a radiometer (Model-470D, Sanso, Tokyo, Japan) and attenuated to equal density (5.0×10^11^ photons cm^−2^ s^−1^) at the corneal surface using an optical wedge. The ERG responses were recorded through a biophysical amplifier (AVB-10, Nihon Kohden, Tokyo, Japan) with a high-impedance probe (JB-101J, Nihon Kohden) connected to a computer with the AcqKnowledge 3.9.1.6 software (BIOPAC Systems, Goleta, CA, USA) via an analogue-to-digital (A/D) converter (MP-150, BIOPAC Systems).

We stimulated the dark-adapted eye with a series of monochromatic flashes of 200 ms duration, separated by 15 s intervals, to record spectral response. The wavelength first varied from short to long wavelengths and then in the reverse order. The pairs of bidirectional stimulation were recorded at least twice. We then measured the responses to light of varying intensities over a 4 log-unit at the wavelength that gave the maximum response. The response amplitude-log intensity (*V*-log *I*) data were fitted to the Naka­­­–Rushton function:
(1)

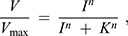
where *I* is the stimulus intensity, *V* is the response amplitude, *V*_max_ is the maximum response amplitude, *K* is the stimulus intensity eliciting 50% of *V*_max_ and *n* is the exponential slope (Naka and Rushton, 1966), using the custom-made programs implemented in MATLAB R2018a (The MathWorks, Inc., Natick, MA, USA). The sensitivity is defined as the reciprocal of the stimulus intensity *I* required for a criterion response (Menzel, 1979). Based on the obtained sigmoidal *V*-log *I* function, we converted the spectral response into the spectral sensitivity (*S*) and normalised it to the maximum sensitivity. We calculated *S* for each individual by averaging the 2–4 paired spectral series from the ERG recording in one sample, and then determined *S* as mean±s.e.m. for each species by averaging those of the multiple individuals.

### Estimation of visual pigment absorption spectra

We assumed that the hawkmoth ERG-determined *S* could be modelled as a weighted sum of the absorption spectra of UV, SW and LW-absorbing visual pigments (Satoh et al., 2017). According to Stavenga et al. (1993) and Stavenga (2010), the absorbance spectrum of the visual pigments (*R_i_*) is assumed as the summation of the α- and β-absorbance bands:
(2)

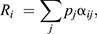
where *i*=UV, SW, LW, *j*=α-band, β-band and *p_j_* is the relative amplitude. α*_ij_* is the absorbance of each band, which is described by:
(3)

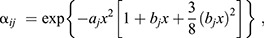
with:
(4)

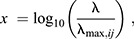
where λ is the wavelength and λ_max,*ij*_ is the peak wavelength (Stavenga et al., 1993). The parameter values are as follows: *p*_β-band_ / *p*_α-band_*=*0.29, *a*_α-band_=380, *b*_α-band_=6.09, *a*_β-band_=247, *b*_β-band_=3.59 (Stavenga et al., 1993). We fixed the λ_max_ of the β-band at 360 nm according to the previous fit in the butterfly photoreceptors (Arikawa et al., 1999).

The simplest case of ERG-determined *S* with three visual pigments can be modelled as a weighted sum of the template absorbance spectra:
(5)

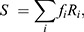
where *f_i_* is the relative contribution. We estimated the parameters λ_max_ and *f_i_* by the model fits using the nonlinear least-squares algorithm nlsLM from the R package ‘minpack.lm’ (https://CRAN.R-project.org/package=minpack.lm).

Satoh et al. (2017) measured and modelled the absorbance spectra of the screening pigments in the summer fruit tortrix moth *Adoxophyes orana*. Following their procedure, we modelled the screening pigment absorbance spectrum, *A*, with a sum of two Gaussian functions:
(6)

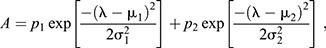
where μ_1_ and μ_2_ are the peak wavelengths, σ_1_ and σ_2_ are the standard deviations (s.d.), and *p*_1_ and *p*_2_ are the relative contributions, respectively. We incorporated *A* in Eqn 5 to estimate the λ_max_ values with the effect of the screening pigments taken into account. The resulting spectral sensitivity *S*_a_ is:
(7)

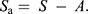


We estimated the parameters λ_max_, *f_i_*, μ_1_, μ_2_, σ_1_, σ_2_, *p*_1_ and *p*_2_ using the nonlinear least-squares algorithm nlsLM from the R package ‘minpack.lm’.

### Histology of compound eye

For anatomy, we used males of the same four species as used for electrophysiology. The eyes were light adapted for 30 min under room light. The light-adapted eyes were dissected under a dissection microscope and pre-fixed in 2% paraformaldehyde and 2.5% glutaraldehyde in 0.1 mol l^−1^ sodium cacodylate buffer (CB) at pH 7.4 for 30–60 min at room temperature. After being washed with 0.2 mol l^−1^ CB, the eyes were dehydrated through an acetone series, infiltrated with propylene oxide, and embedded in Epon. We cut 10 μm thick sections with a rotatory microtome (HM 355 S, MICROM GmbH, Walldorf, Germany) and observed the sections under a light microscope (BX60, Olympus Optical Co., LTD, Tokyo, Japan) without staining.

## RESULTS

### Three independent emergences of diurnal lineages in hawkmoths

We constructed an ML tree based on the RNA-seq short-read sequences to investigate the phylogeny of hawkmoth species (Fig. 1A). The phylogenetic tree inferred from 17,305 informative positions representing the whole genome can be considered as a hawkmoth species tree. All branches were supported by high bootstrap values (97–100), except for one. The phylogenetic relationship was almost the same as that proposed in a previous study based on five nuclear loci (Kawahara et al., 2009). We estimated the emergence of diurnal species/lineages based on the species tree, assuming that diurnal species diverged from the nocturnal ancestors and the evolution in the opposite direction did not occur because most species/lineages of hawkmoths are nocturnal and the nocturnal niche has been already occupied by ancestral hawkmoth species. As shown in Fig. 1A, the basal lineage of hawkmoth species was nocturnal, which has been suggested by Kawahara et al. (2009). The diurnal species/lineages independently appeared three times: the *C. hylas/H. affinis*, *N. himachala* and *M. pyrrhosticta/M. bombylans* lineages, named as D1, D2 and D3, respectively (D1–D3 in Fig. 1A).

### Expression of opsin genes

We assembled the transcriptomes which consistently contained 85.3–94.7% of complete and fragmented BUSCOs, and identified three visual opsin genes (*UV*, *SW* and *LW*) in the transcriptomes of all 10 hawkmoth species. Subsequently, we assembled both ends of the coding regions with UTRs and verified the full-length cDNA sequences by PCR and sequencing.

**Fig. 2.**
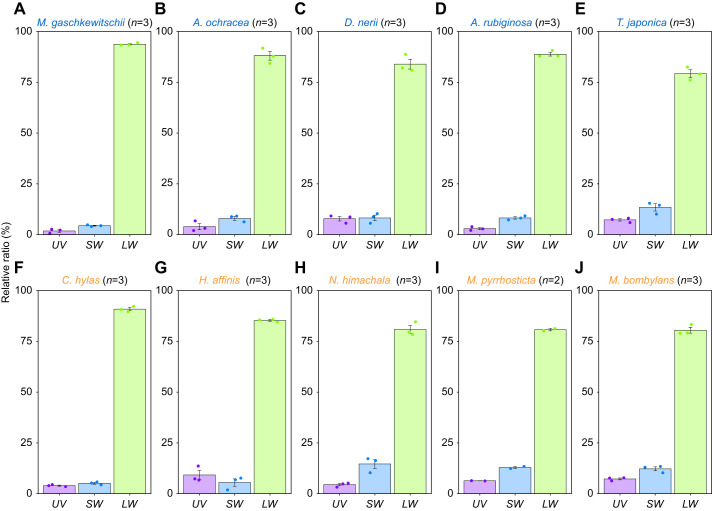
**Relative expression ratios of the three opsin genes in 10 hawmoth species.** Relative mean±s.e. expression ratios of mRNAs encoding the three opsins in (A–E) nocturnal and (F–J) diurnal hawkmoth species were quantified based on RPKM values. A dot represents the ratio in one individual, and the number of individuals (*n*) is shown next to the species name.

We then performed expression analysis for the three opsin genes in the 10 species (Fig. 2). The relative expression ratios were 1.8–9.2% in *UV*, 4.4–14.6% in *SW* and 79.3–93.8% in *LW* genes. The relative expression levels of the *SW* gene appeared to be slightly higher than that of the *UV* gene, but the order was reversed in *H. affinis* (Fig. 2). These results suggest that the *LW* gene is predominantly expressed, while the *UV* and *SW* genes are expressed almost equally in hawkmoth eyes.

We compared the RPKM values of these genes between the nocturnal and diurnal species and found no significant differences in the expression values between the nocturnal and diurnal species for all the three opsin genes (*P*>0.15, in every case).

### Parallel evolution of opsin genes in diurnal lineages

Fig. 1B–D shows the ML gene trees of the three opsin genes. The topology of the UV opsin tree (Fig. 1B) reflects the species' phylogenetic relationships (Fig. 1A). In contrast, the SW and LW opsin trees exhibit distinct branching patterns from the species tree: SW and LW opsins in diurnal species form monophyletic groups in both trees (Fig. 1C,D). In the AU test comparing the ML gene tree to the gene tree with the species tree topology, the ML tree of UV opsin was not significantly different from the other (*P*=0.69), whereas the ML trees of SW and LW opsins were significantly supported (*P*=0.99 and *P*=0.99, respectively). These results indicate that the parallel amino acid substitutions may have occurred on the diurnal branches under selective pressure different from those on the nocturnal branches (Kreitman and Akashi, 1995; Adachi and Hasegawa, 1996). Therefore, we first estimated and compared the dN/dS ratio (ω value) to ascertain whether the amino acid substitutions have accelerated in the diurnal branches.

The ω values of the branches are <1 in all the opsins, which means that the three visual opsin genes have evolved under negative (purifying) selection with functional constraints on the entire gene throughout their evolution (one-ratio model; Table 1). However, we found that the ω values of the diurnal branches were significantly higher than those of the nocturnal branches in all three opsins (*P*<0.05, two-ratio model; Table 1). In the branch model comparison, if the ω value on the foreground diurnal branch is higher than that on the background nocturnal branch but not greater than 1, the result is not sufficient to suggest that positive selection has acted on the gene. Then, we extended the analysis to see whether non-neutral parallel evolution has occurred on the diurnal opsin genes.

**Table 1.**
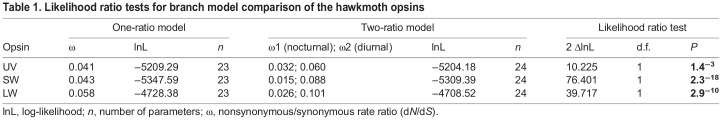
Likelihood ratio tests for branch model comparison of the hawkmoth opsins

**Fig. 3.**
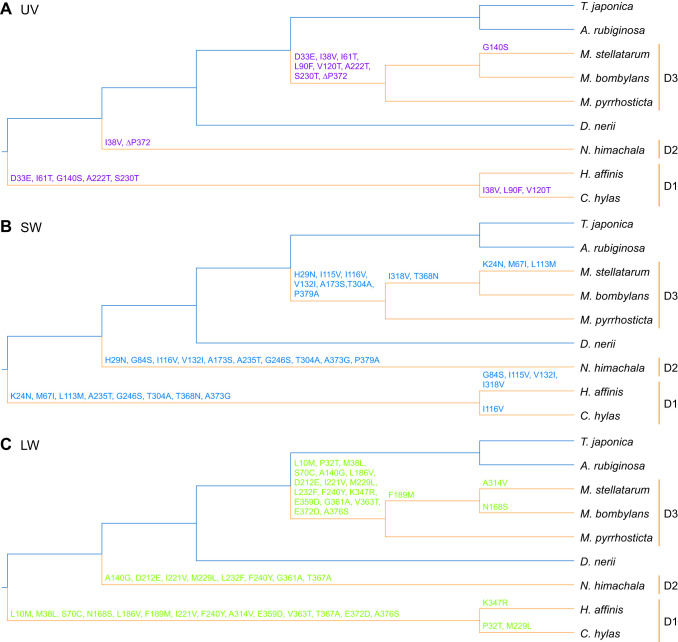
**Parallel amino acid substitutions of the opsins on the diurnal branches.** The tree topology is based on the phylogenetic tree of hawkmoth species (Fig. 1A). The *M. stellatarum* branch is joined with the *M. bombylans* branch on the basis of the three opsin gene trees (Fig. 1B–D). The parallel amino acid substitutions in (A) UV, (B) SW and (C) LW opsins among the diurnal species/lineages are shown on the branches. The indigo branches are nocturnal, and the orange branches are diurnal. D1–D3 represent diurnal clades. The amino acid residue numbers follow those of each orthologous opsin in *M. sexta*.

We inferred the ancestral amino acid sequences at each node, and identified the parallel amino acid substitutions in at least two of the three diurnal lineages (D1–D3 in Fig. 3). The total numbers of amino acid substitutions which occurred in parallel were 8 for UV opsin, 16 for SW opsin and 21 for LW opsin (Fig. 3; Fig. S1, Table 2). We also identified one parallel deletion (ΔP372) in two diurnal branches – D2 and D3 – of UV opsin (Fig. 3A; Fig. S1A). A pairwise statistical analysis was then performed to compare the number of parallel amino acid substitutions with the expected number which would occur by chance under neutral evolution. For SW and LW opsins, the numbers of parallel substitutions observed in all 11 pairs of diurnal lineages were significantly larger than expected (*P*<0.05, Table 2). The observed numbers for UV opsin were significantly larger than expected in 8 of the 11 pairs of diurnal lineages (*P*<0.05, in those cases; Table 2).

**Table 2.**
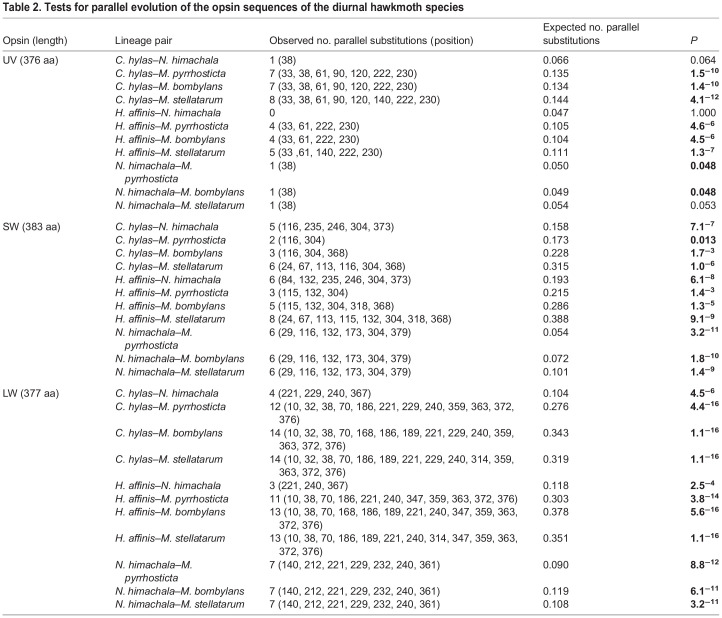
Tests for parallel evolution of the opsin sequences of the diurnal hawkmoth species

### Absorption spectra of visual pigments

**Fig. 4.**
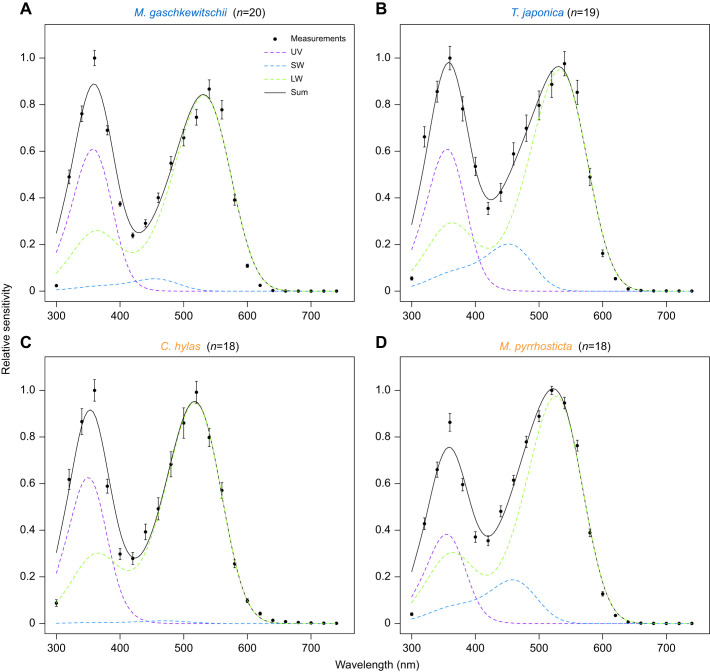
**Spectral sensitivities of the compound eyes determined by ERG and the estimated visual pigment absorption spectra.** In the (A,B) nocturnal and (C,D) diurnal species, filled circles indicate mean±s.e.m. measurements in multiple individuals (*n*) shown next to the species name. Dashed coloured lines are the absorption spectra of the individual visual pigments, and the solid black line is the weighted sum of the three spectra.

To assess the absorption spectra of the three visual pigments, we measured the eye's spectral sensitivity, *S*, by recording ERG responses to monochromatic lights. Fig. 4 shows the *S* of the dark-adapted hawkmoths (filled circles with error bars). All spectra exhibit peaks in the UV and green wavelength regions, typical in many insects including moths (Wakakuwa et al., 2014; Satoh et al., 2017). The dotted coloured lines in each panel represent the predicted absorption spectra of UV, SW and LW pigments multiplied by the contribution factors (*f_i_* in Eqn 5). The contribution of LW pigments is equally high in all the species. The solid black line is the summation of these three spectra.

We revealed that the predicted absorption spectra of SW and LW pigments differed between the nocturnal and diurnal species. Table 3 summarises the estimated λ_max_ and *f_i_* values. In the diurnal species, the λ_max_ values of SW pigments shifted about 10 nm on average to the long-wavelength direction: 16.0 nm shift for *C. hylas* and 5.0 nm shift for *M. pyrrhosticta* from the average value of nocturnal species, respectively. On the other hand, the shift was in the opposite direction in LW pigments: the λ_max_ values were about 10 nm shorter in diurnal species with a 14.7 nm shift for *C. hylas* and 5.8 nm shift for *M. pyrrhosticta* from the average value of nocturnal species, respectively. However, the λ_max_ values of UV pigments were similar.

**Table 3.**
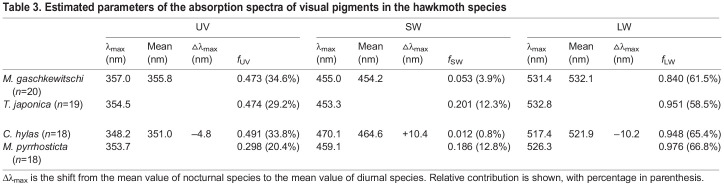
Estimated parameters of the absorption spectra of visual pigments in the hawkmoth species

## DISCUSSION

### Driving force for parallel evolution of diurnal opsin sequences

In this study, we constructed a phylogenetic tree of hawkmoth species and show that the diurnal lineages of hawkmoths independently appeared at least three times in the subfamily Macroglossinae after hawkmoth divergence around 42.8 MYA before subtribe Choerocampina divergence around 12.2 MYA (Kawahara et al., 2019). Opsin gene duplications and losses have occurred frequently in animals, including lepidopteran insects. For example, diurnal butterflies have more opsin genes than nocturnal moths, which, at least in part, explains the spectral richness in butterfly eyes (van der Kooi et al., 2021). However, the hawkmoths were found to switch their ecology from nocturnal to diurnal while keeping their opsin gene repertoire. To the best of our knowledge, the evolutionary process within a fixed set of opsins has never been properly addressed in the context of the diurnal–nocturnal transition. The present study on the hawkmoth opsins is unique in that it enables us to compare the opsin gene sequences and predicted opsin spectral sensitivities independently of the differences in gene number.

Why has gene duplication not occurred in hawkmoths? Gene duplication occurs mainly through recombination when two genes with similar sequences are located close to each other. In the *M. sexta* genome, the *LW* gene is located alone on chromosome 28. The *UV* and *SW* genes are located a long distance apart on chromosome 15. The relationship between these two genes is widely conserved in other lepidopteran insects in which these genes are not duplicated (Gershman et al., 2021). Therefore, it is presumed that opsin gene duplication has been unlikely to occur in hawkmoths owing to their genome structure, even though they have experienced light environment changes.

In addition to the opsin gene repertoire, we found no difference in the expression patterns of the three opsin genes between the diurnal and nocturnal hawkmoth species, while the evolutionary rates of amino acid substitutions differed significantly between them, although all ω values were below 1 (Table 1). Furthermore, many parallel amino acid substitutions between the different diurnal lineages were observed in the three opsins (Fig. 3; Fig. S1, Table 2). These parallel substitutions exceeded the numbers expected under neutral evolution, suggesting that parallel evolution of the opsin sequences in diurnal species has been driven by positive selection. In the hawkmoths, the repertoire of opsin genes and the expression patterns of those genes have certainly been functionally constrained, preventing them from changing during the ecological transition from nocturnal to diurnal vision. Instead, the amino acid sequences of opsins would have been substituted by positive selection during the transitions, resulting in a large number of parallel amino acid substitutions. Although the effect of each parallel amino acid substitution on the function of the visual pigments is not known, the parallel substitutions possibly cause a functional change that is beneficial in the daylight environment.

### Spectral shifts of visual pigments in diurnal–nocturnal transitions

We predicted the absorption spectra of the visual pigments based on the ERG-determined spectral sensitivities (Fig. 4, Table 3). The λ_max_ values roughly match with those reported previously in the hawkmoths (van der Kooi et al., 2021). Determination of visual pigment absorption spectra is often tricky. Any of the currently available methods, such as ERG recording, single-cell electrophysiology, microspectrophotometry and *in vitro* expression, has its own strength and weakness. Here, we employed ERG recording, which could have been accompanied by selective adaptation using monochromatic lights (Telles et al., 2014). However, we decided not to take this approach because even weak monochromatic lights can cause light adaptation of eyes presumably due to screening pigment migration which often makes the responses undetectable, particularly in the nocturnal species. Instead, we took the approach of increasing the number of samples (18–20 individuals for each species; Fig. 4, Table 3), where we applied identical methods to all the individuals in both the nocturnal and diurnal species. The resulting spectral sensitivities were quite stable with small variations and therefore the predicted visual pigment spectra were also steady. The results indicate that in two diurnal species, the peak absorption wavelength, λ_max_, of *C. hylas* and *M. pyrrhosticta* shifts 16 nm and 5 nm in SW pigments towards long wavelength direction, and 15 nm and 6 nm in LW pigments towards the short wavelength direction, respectively.

Still, the model calculations deviate considerably, particularly in the UV region, and the contributions of SW pigments to the spectral sensitivities are estimated to be small (Fig. 4). The difficult estimation is most likely due to the filtering effect of the screening pigments, which also exist in hawkmoth eyes (Fig. S2). We could actually obtain better fits by incorporating presumptive pigment absorption spectra in the model. Even in the calculation with the function of the screening pigments, the predicted λ_max_ values are close to those without the pigments (Table S3).

**Fig. 5.**
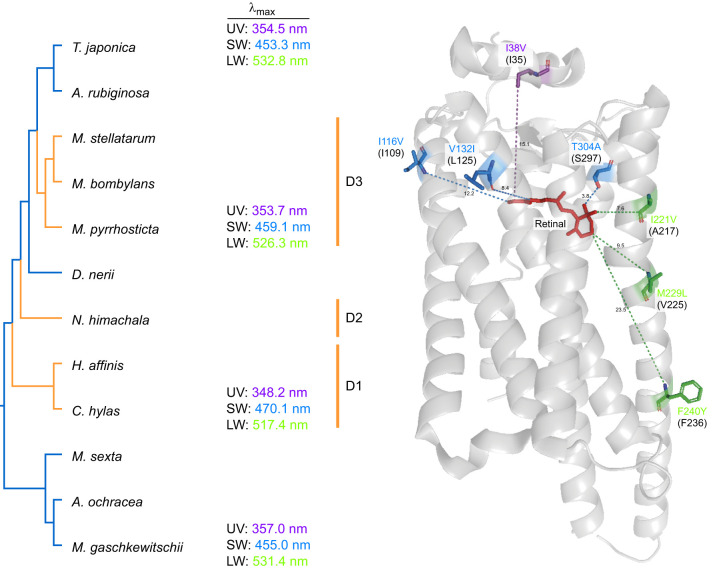
**Common parallel substitutions among the three diurnal branches and absorption spectra of the opsin visual pigments.** The tree topology is based on the phylogenetic tree of hawkmoth species (Fig. 1A). The indigo and orange branches are nocturnal and diurnal, respectively. The λ_max_ values of the three visual pigments estimated in this study are shown on the right side of the species names. For the three opsins, the common parallel amino acid substitutions occurring in the three diurnal branches are shown on the right side of the diurnal clade names D1–D3 and those positions are indicated on the 3D model of jumping spider Rh1 opsin. The minimum distances (Å) between the retinal chromophore (red) and the amino acid positions are depicted by dotted coloured lines. Corresponding amino acid positions in jumping spider Rh1 are shown in the parentheses under the amino acid substitutions. The colours of the λ_max_ values and the substitutions represent the three opsin pigments: violet, UV pigment; blue, SW pigment; green, LW pigment. Amino acid residue numbers for the substitutions follow those of each orthologous opsin in *M. sexta*.

The shifts in the λ_max_ values are presumably attributed to the detected parallel amino acid substitutions. Fig. 5 presents the common parallel substitutions in all the three diurnal lineages (D1–D3): one for UV (I38V) and three for SW (I116V, V132I, T304A) and LW (I221V, M229L, F240Y) opsins. The probability of the parallel substitution occurring independently under neutral evolution in three lineages should be much smaller than in two lineages (Zhang and Kumar, 1997). There may be some synergistic effects of the amino acid substitutions, which would explain why some sites are far from the retinal chromophore (Fig. 5). At any rate, these sites are the likely candidates for focusing future investigation of the molecular mechanism underlying the spectral shifts.

### Relationship between hawkmoth vision and light environments

The visual pigments mainly function in the eyes. An insect's compound eye consists of thousands of small units called ommatidia, each typically containing eight or nine photoreceptor cells. The photoreceptor cells together form a visual pigment-containing rhabdom. The visual pigments absorb the light propagating in the rhabdom and trigger the phototransduction cascade, which eventually depolarises the photoreceptor membrane. Although examples of co-expression of opsin genes have been reported in insects (Arikawa and Stavenga, 2014), one photoreceptor is typically assumed to express one type of opsin corresponding to a particular visual pigment, whose absorption spectrum principally determines the photoreceptor's spectral sensitivity. Accumulated evidence indicates that an ommatidium generally bears seven LW opsin-expressing photoreceptor cells and two UV or SW opsin-expressing cells (van der Kooi et al., 2021). Hence, the ratio roughly matches the opsin gene expression ratio we found in the hawkmoths (Fig. 2). Note also that there are three possible combinations of UV or SW opsin-expressing cells: two UVs, two SWs, or one of each, making the ommatidia spectrally heterogeneous. These three types of ommatidia randomly fill the hexagonal lattice at least locally (Arikawa and Stavenga, 2014). The basic spectral organisation of ommatidia and their random array also hold for the Tobacco hawkmoth *M sexta* (White et al., 2003).

The LW opsin-expressing photoreceptors are green sensitive in most cases. However, some of the LW opsin-expressing cells are red sensitive in the ommatidia of pierid butterflies, where the dense reddish pigments surround the rhabdom (Stavenga and Arikawa, 2011). The perirhabdomal pigments alter the spectral contents of the light propagating in the thin rhabdom by absorbing the boundary wave, thus changing the cell's spectral sensitivity. In the hawkmoth eyes, we did not find any sign of such perirhabdomal pigments (Fig. S2). Plus, the diameter of the hawkmoths' rhabdom is large (Warrant et al., 1999), which prevents any perirhabdomal pigments from functioning as a robust spectral filter. Therefore, the absence of perirhabdomal pigments suggests that LW opsin-expressing cells in hawkmoth eyes are most likely to be green sensitive.

Green receptors are the most numerous and they localise in all ommatidia making a complete hexagonal lattice in the compound eye. In contrast, the distribution of UV and blue receptors has gaps because of the random array. The complete hexagonal lattice of the green receptor system is presumably crucial in achromatic spatial and motion vision (Yamaguchi et al., 2008; Stewart et al., 2015). The green receptors also contribute to colour vision (von Helversen, 1972; Koshitaka et al., 2008) and UV–blue–green trichromacy has been proposed in the Hummingbird hawkmoth *M. stellatarum* (Telles et al., 2016).

Parallel evolution in diurnal hawkmoth opsin sequences may have been caused by positive selection pressures most likely to be adaptive for daytime activity. Compared with the nocturnal *D. elpenor*, the diurnal *M. stellatarum* relies more on visual cues than olfactory ones (Balkenius et al., 2006; Stöckl et al., 2016). As described above, the λ_max_ values of LW and SW pigments in the diurnal species shifted 10 nm in the short and long wavelength direction, respectively meaning that the separation of the sensitivity spectra of these cells has become 20 nm smaller. For colour vision, a 20 nm difference in the photoreceptor spectral sensitivity is not negligible: human colour deficiency can be attributed to only several nanometre differences in the cone spectral sensitivity (Bowmaker, 1998) and *M. stellatarum* with trichromatic vision discriminates the wavelength differences of 1–2 nm at around 380 nm and 480 nm, which correspond to the wavelength regions where the spectral sensitivities of the photoreceptors overlap (Telles et al., 2016). The *Papilio* butterfly with tetrachromatic vision also discriminates the wavelength differences of a few nanometres (Koshitaka et al., 2008). The large separation between λ_max_ values of the visual pigments in the nocturnal species widens the visible spectral range, which could gain absolute sensitivity in a light-limited environment (Johnsen et al., 2006). However, the reduced λ_max_ separation increases the overlap of the spectral sensitivities, which potentially enhances the wavelength discrimination that the diurnal species can utilise. The reduced λ_max_ separation has also been recently reported in *Heliconius* butterflies, which have lost the UV2 receptor, and in *Heliconius ismenius*, the λ_max_ values of the UV1 and blue receptors are shifted toward longer and shorter wavelengths, respectively (McCulloch et al., 2022). This reduced λ_max_ separation in *H. ismenius* may enhance colour discrimination in the short wavelength range owing to a greater overlap of the spectral sensitivities between the UV1 and blue receptors compared with that of *Heliconius melpomene*. In two diurnal hawkmoths, the separation between λ_max_ values of the visual pigments were different: 47 nm in *C. hylas* and 67 nm in *M. pyrrhosticta*. The difference in these values may be related to differences in wavelength discrimination ability, suggesting that *C. hylas* has higher discrimination ability than that *M. pyrrhosticta*.

Because we focused on determining the absorption spectra of the visual pigments, we did not extend the analysis to dorso-ventral specialisation. Nevertheless, we have indeed noticed that the ventral eye region of *C. hylas* was almost exclusively sensitive to UV (Fig. S2C), which seems unique to this species. Inverted cases are known in other insects, including *M. sexta* where more UV opsin-expressing photoreceptors are distributed in the dorsal region of the eye (White et al., 2003), which is consistent with the dorsal region being more sensitive to UV (Bennett et al., 1997). In the diurnal owl-fly *Libelloides macaronius*, for instance, the dorsal region exclusively sensitive to UV is optimal for owl-flies detecting small flying targets against the bright sky (Belušič et al., 2013).

### Conclusions

In the current study of nocturnal and diurnal hawkmoths, we identified the opsin genes, their expression levels and possible absorption spectra of visual pigments. The transitions from nocturnal to diurnal ecology in hawkmoths were accompanied by parallel amino acid substitutions in visual opsins, which presumably brought spectral sensitivities of LW and SW pigments closer and enhanced their colour discrimination properties. Besides the shifts in spectral sensitivities that we revealed, other visual phenotypes [e.g. the numbers of the facets, the morphologies of the lamina monopolar cells (LMCs) in the optic lobe, and the responses of the motion-sensitive neurons in the lobula complex] also differ between the nocturnal and diurnal hawkmoth species (Stöckl et al., 2017). More detailed analyses of the anatomical distribution of visual pigments, the physiological properties of eyes and associated visual behaviour will further shed light on the adaptive molecular evolution in the opsin genes and other genes of hawkmoths through their diurnal–nocturnal transition.

## Supplementary Material

10.1242/jexbio.244541_sup1 Supplementary information
