# Supplementary material for: Parallel evolution of opsin visual pigments in hawkmoths by tuning of spectral sensitivities during transition from a nocturnal to a diurnal ecology
Source: J Exp Biol. 2022 Dec 12;225(23):jeb244541. doi: 10.1242/jeb.244541 (PMC10112871; doi:10.1242/jeb.244541)
Supplement: Supplementary information [file jexbio-225-244541-s1.pdf]

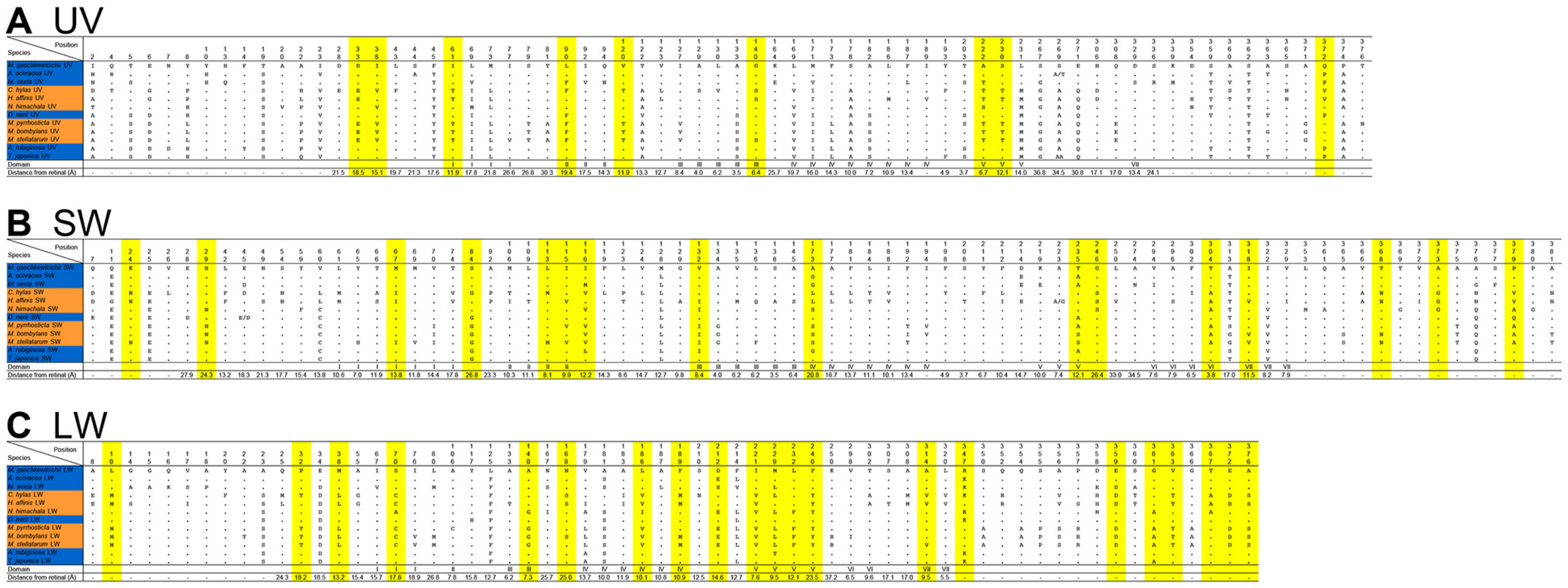

Fig. S1. Amino acid alignments for the three opsins. Amino acid alignments of the informative positions for (A) UV, (B) SW, and (C) LW opsins. The parallel substitutions occurring in the diurnal species/lineages are highlighted in yellow. The amino acid positions follow those of each orthologous opsin in *M. sexta*. Dots indicate identical residues to those of the top line. The transmembrane domains were deduced based on each orthologous opsin sequence of *M. sexta,* and the minimum distances between the retinal and the amino acid positions were estimated based on the 3D structure of jumping spider Rh1.

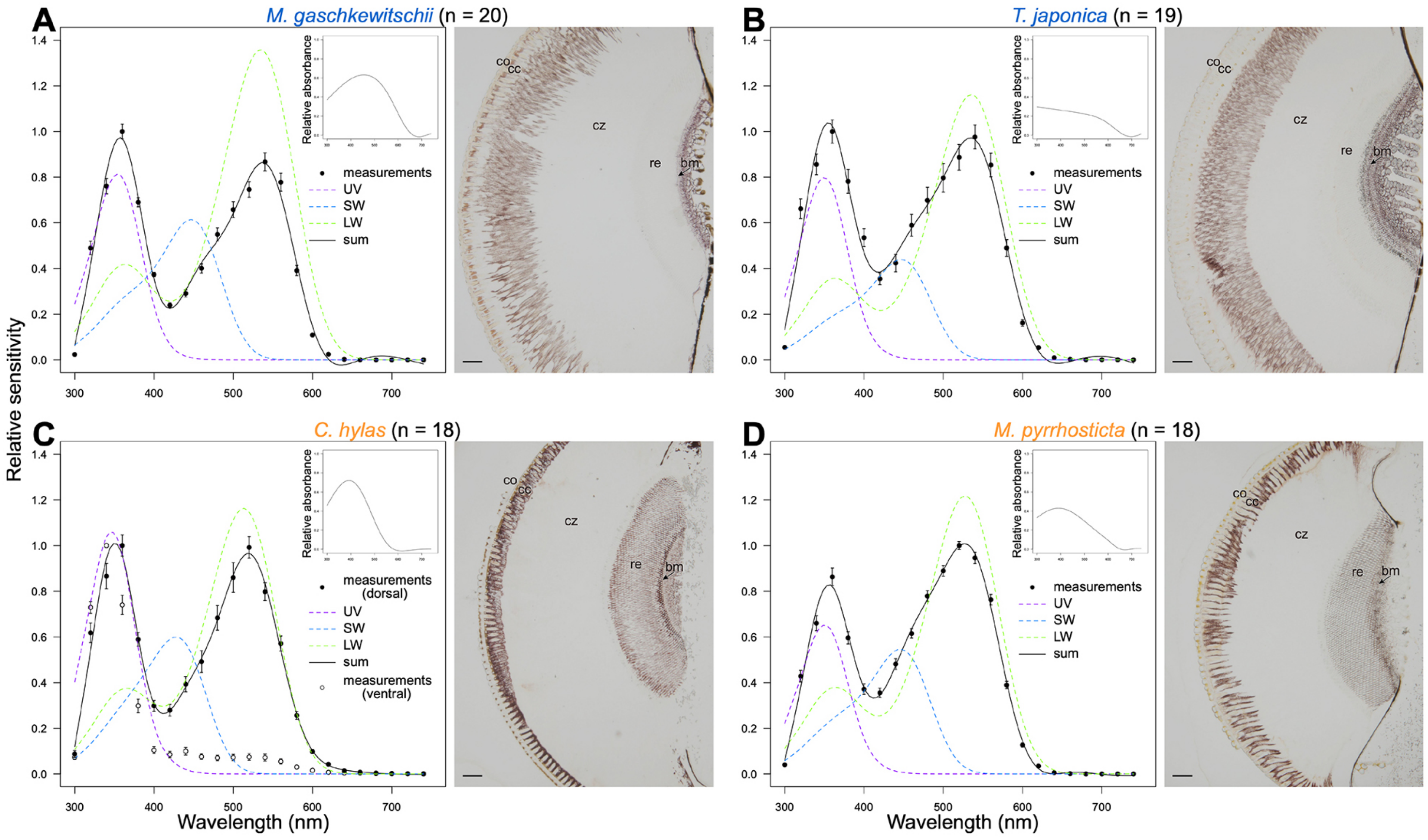

Fig. S2. Spectral sensitivities of the compound eyes determined by ERG and the absorption spectra of visual pigments estimated with the effect of screening pigments. In the (A, B) nocturnal and (C, D) diurnal species, filled circles indicate mean ± SE of the measurements in multiple individuals, n, shown next to the species name. The open circles in panel C indicate the recorded sensitivity from the ventral eye region of *C. hylas* (n = 20: 16 males and 4 females). Dotted color lines are the absorption spectra of the individual visual pigments in the model fitting with the effect of the screening pigments. Solid line is the weighted sum of the three spectra minus the absorption spectrum of the screening pigments. Inset shows the spectrum of presumptive screening pigments. A frontal section of the compound eye is shown next to the spectral sensitivity. The clear zone (cz) separates the dioptric apparatus (crystalline cone, cc, and cornea, co) and the retina (re), where screening pigments reside. Arrow points to the position of the basement membrane (bm). Scale bar = 100 µm.

The model fitting to the ERG data indicated that the contribution of UV and SW pigments were equally high, while LW pigments contributed most (Table 3; Table S3). The results fitted even better to the results of the expression ratios of the opsin genes (Fig. 2). The estimated λ_{max} values of UV and LW pigments were also similar: importantly, the shift direction and the amount both in LW pigments appear robust (Table 3; Table S3). On the other hand, for the estimated λ_{max} values of SW pigments the shift direction was opposite (Table 3; Table S3). This contradictory result is probably led by an inaccurate estimation due to the overlap of the absorption peak of SW pigments and the Gaussian mound in the screening pigments' absorption spectrum. Thus, measurement of the absorbance spectra of the screening pigments in the hawkmoths is warranted in the futu

Journal of Experimental Biology • Supplementary information

Table S1. Hawkmoth sample information surveyed by RNA-seq

Sample ID	Species	Subfamily	Sampling location	Sampling date	Sampling method	Collector	Sex	Ecology	Tissues	Fixation date & time	No. of reads	Experiment accession no.	Run accession no.
Mga1	*Marumba gaschkewitschii echephron* (Boisduval, 1875)	Smerinthinae	Yokosuka, Kanagawa, Japan (35°15'N, 139°37'E)	28/07/2014	Adult collection	T.A.	Male	Nocturnal	Adult eyes & brain	31/07/2014 12:00-13:00	27,175,728	DRX229139	DRR239257
Mga2	*Marumba gaschkewitschii echephron* (Boisduval, 1875)	Smerinthinae	Yokosuka, Kanagawa, Japan (35°15'N, 139°37'E)	28/07/2014	Adult collection	T.A.	Male	Nocturnal	Adult eyes & brain	01/08/2014 12:00-13:00	31,977,640	DRX229140	DRR239258
Mga3	*Marumba gaschkewitschii echephron* (Boisduval, 1875)	Smerinthinae	Yokosuka, Kanagawa, Japan (35°15'N, 139°37'E)	28/07/2014	Adult collection	T.A.	Male	Nocturnal	Adult eyes & brain	01/08/2014 12:00-13:00	30,222,302	DRX229141	DRR239259
Aoc1	*Ambulyx ochracea* Butler, 1885	Smerinthinae	Yokosuka, Kanagawa, Japan (35°15'N, 139°37'E)	21/07/2014	Adult collection	T.A.	Male	Nocturnal	Adult eyes & brain	24/07/2014 12:00-13:00	33,952,206	DRX229142	DRR239260
Aoc2	*Ambulyx ochracea* Butler, 1885	Smerinthinae	Yokosuka, Kanagawa, Japan (35°15'N, 139°37'E)	24/07/2014	Adult collection	T.A.	Male	Nocturnal	Adult eyes & brain	29/07/2014 12:00-13:00	28,472,930	DRX229143	DRR239261
Aoc3	*Ambulyx ochracea* Butler, 1885	Smerinthinae	Yokosuka, Kanagawa, Japan (35°15'N, 139°37'E)	28/07/2014	Adult collection	T.A.	Male	Nocturnal	Adult eyes & brain	31/07/2014 12:00-13:00	28,297,454	DRX229144	DRR239262
Chy1	*Cephonodes hylas hylas* (Linnaeus, 1771)	Macroglossinae	Higashikurume, Tokyo, Japan (35°45'N, 139°32'E)	31/08/2014	Adult collection	T.A.	Male	Diurnal	Adult eyes & brain	01/09/2014 12:00-13:00	19,708,896	DRX229145	DRR239263
Chy2	*Cephonodes hylas hylas* (Linnaeus, 1771)	Macroglossinae	Higashikurume, Tokyo, Japan (35°45'N, 139°32'E)	13/09/2015	Adult collection	T.A.	Male	Diurnal	Adult eyes & brain	14/09/2015 12:00-13:00	27,273,782	DRX229146	DRR239264
Chy3	*Cephonodes hylas hylas* (Linnaeus, 1771)	Macroglossinae	Higashikurume, Tokyo, Japan (35°45'N, 139°32'E)	14/09/2015	Adult collection	T.A.	Male	Diurnal	Adult eyes & brain	15/09/2014 12:00-13:00	28,864,550	DRX229147	DRR239265
Haf1	*Hemaris affinis* (Bremer, 1861)	Macroglossinae	Hayama, Kanagawa, Japan (35°15'N, 139°36'E)	06/08/2014	Adult collection	T.A.	Male	Diurnal	Adult eyes & brain	07/08/2014 12:00-13:00	31,536,052	DRX229148	DRR239266
Haf2	*Hemaris affinis* (Bremer, 1861)	Macroglossinae	Hayama, Kanagawa, Japan (35°15'N, 139°36'E)	02/09/2014	Adult collection	T.A.	Male	Diurnal	Adult eyes & brain	03/09/2014 12:00-13:00	29,844,236	DRX229149	DRR239267
Haf3	*Hemaris affinis* (Bremer, 1861)	Macroglossinae	Hayama, Kanagawa, Japan (35°15'N, 139°36'E)	06/09/2015	Adult collection	T.A.	Male	Diurnal	Adult eyes & brain	07/09/2015 12:00-13:00	31,834,506	DRX229150	DRR239268
Nhi1	*Neogurelca himachala sangaica* (Butler, 1876)	Macroglossinae	Yokosuka, Kanagawa, Japan (35°15'N, 139°37'E)	05/08/2014	Adult collection	T.A.	Male	Diurnal	Adult eyes & brain	06/08/2014 12:00-13:00	30,722,512	DRX229151	DRR239269
Nhi2	*Neogurelca himachala sangaica* (Butler, 1876)	Macroglossinae	Higashikurume, Tokyo, Japan (35°45'N, 139°32'E)	31/08/2015	Adult collection	T.A.	Female	Diurnal	Adult eyes & brain	01/09/2015 12:00-13:00	26,655,558	DRX229152	DRR239270
Nhi3	*Neogurelca himachala sangaica* (Butler, 1876)	Macroglossinae	Higashikurume, Tokyo, Japan (35°45'N, 139°32'E)	14/09/2015	Adult collection	T.A.	Female	Diurnal	Adult eyes & brain	15/09/2015 12:00-13:00	29,837,426	DRX229153	DRR239271
Dne1	*Daphnis nerii* (Linnaeus, 1758)	Macroglossinae	Ishigaki, Okinawa, Japan (24°20'N, 124°10'E)	27/09/2014	Larvae rearing	T.A.	Female	Nocturnal	Adult eyes & brain	14/10/2014 12:00-13:00	24,244,384	DRX229154	DRR239272
Dne2	*Daphnis nerii* (Linnaeus, 1758)	Macroglossinae	Ishigaki, Okinawa, Japan (24°20'N, 124°10'E)	27/09/2014	Larvae rearing	T.A.	Female	Nocturnal	Adult eyes & brain	19/10/2014 12:00-13:00	23,958,174	DRX229155	DRR239273
Dne3	*Daphnis nerii* (Linnaeus, 1758)	Macroglossinae	Ishigaki, Okinawa, Japan (24°20'N, 124°10'E)	27/09/2014	Larvae rearing	T.A.	Female	Nocturnal	Adult eyes & brain	20/10/2014 12:00-13:00	25,434,320	DRX229156	DRR239274
Mpy1	*Macroglossum pyrrhosticta* Butler, 1875	Macroglossinae	Hayama, Kanagawa, Japan (35°15'N, 139°36'E)	31/08/2015	Adult collection	T.A.	Male	Diurnal	Adult eyes & brain	01/09/2015 12:00-13:00	30,885,830	DRX229157	DRR239275
Mpy2	*Macroglossum pyrrhosticta* Butler, 1875	Macroglossinae	Hayama, Kanagawa, Japan (35°15'N, 139°36'E)	01/09/2015	Adult collection	T.A.	Male	Diurnal	Adult eyes & brain	02/09/2015 12:00-13:00	36,915,156	DRX229158	DRR239276
Mbo1	*Macroglossum bombylans* Boisduval, 1875	Macroglossinae	Yokosuka, Kanagawa, Japan (35°15'N, 139°37'E)	04/08/2014	Adult collection	T.A.	Female	Diurnal	Adult eyes & brain	05/08/2014 12:00-13:00	22,893,430	DRX229159	DRR239277
Mbo2	*Macroglossum bombylans* Boisduval, 1875	Macroglossinae	Hayama, Kanagawa, Japan (35°15'N, 139°36'E)	11/09/2015	Adult collection	T.A.	Male	Diurnal	Adult eyes & brain	12/09/2015 12:00-13:00	25,896,542	DRX229160	DRR239278
Mbo3	*Macroglossum bombylans* Boisduval, 1875	Macroglossinae	Hayama, Kanagawa, Japan (35°15'N, 139°36'E)	15/09/2015	Adult collection	T.A.	Male	Diurnal	Adult eyes & brain	16/09/2015 12:00-13:00	27,479,356	DRX229161	DRR239279
Aru1	*Ampelophaga rubiginosa rubiginosa* Bremer & Grey, 1853	Macroglossinae	Yokosuka, Kanagawa, Japan (35°15'N, 139°37'E)	21/07/2014	Adult collection	T.A.	Male	Nocturnal	Adult eyes & brain	23/07/2014 12:00-13:00	32,037,438	DRX229162	DRR239280
Aru2	*Ampelophaga rubiginosa rubiginosa* Bremer & Grey, 1853	Macroglossinae	Yokosuka, Kanagawa, Japan (35°15'N, 139°37'E)	21/07/2014	Adult collection	T.A.	Male	Nocturnal	Adult eyes & brain	23/07/2014 12:00-13:00	22,331,642	DRX229163	DRR239281
Aru3	*Ampelophaga rubiginosa rubiginosa* Bremer & Grey, 1853	Macroglossinae	Yokosuka, Kanagawa, Japan (35°15'N, 139°37'E)	28/07/2014	Adult collection	T.A.	Male	Nocturnal	Adult eyes & brain	02/08/2014 12:00-13:00	24,930,292	DRX229164	DRR239282
Tja1	*Theretra japonica* (Boisduval, 1869)	Macroglossinae	Yokosuka, Kanagawa, Japan (35°14'N, 139°35'E)	22/07/2014	Adult collection	T.A.	Male	Nocturnal	Adult eyes & brain	25/07/2014 12:00-13:00	27,381,900	DRX229165	DRR239283
Tja2	*Theretra japonica* (Boisduval, 1869)	Macroglossinae	Yokosuka, Kanagawa, Japan (35°14'N, 139°35'E)	22/07/2014	Adult collection	T.A.	Female	Nocturnal	Adult eyes & brain	25/07/2014 12:00-13:00	33,482,238	DRX229166	DRR239284
Tja3	*Theretra japonica* (Boisduval, 1869)	Macroglossinae	Yokosuka, Kanagawa, Japan (35°14'N, 139°35'E)	25/07/2014	Adult collection	T.A.	Male	Nocturnal	Adult eyes & brain	29/07/2014 12:00-13:00	30,437,216	DRX229167	DRR239285

Table S2. List of primers for PCR and sequencing

Gene	Species	Purpose of use		Primer name	Length	Sequences (5' -> 3')
UV	*M. gaschkewitschii*	PCR	External forward	*HUV*_F01	24	AATGGGCCCAGGAGCACTTCACTG
		PCR	External reverse	*HUV*_R01	24	TTAGCAGGTGCAGCCATAGTTGTC
	A. ochracea	PCR	External forward	*HUV*_F02	24	TCCCACAGACAGAAAAAGCTTCCC
		PCR	External reverse	*HUV*_R02	24	CCATTAGAATAGCCATAGTTGTCG
	C. hylas	PCR	External forward	*HUV*_F03	22	GACTGCTCACGTATCCTACAAC
		PCR	External reverse	*HUV*_R03	25	CCATTAAAAAGCCATGGTTGTCGTC
	H. affinis	PCR	External forward	*HUV*_F04	22	GACTGCTCACGTATCCTTCAAC
		PCR	External reverse	Same with *HUV*_R03		
	N. himachala	PCR	External forward	*HUV*_F05	21	ACTCTACTGAGGACTGCTCAC
		PCR	External reverse	*HUV*_R04	24	GTTTAGAGCGTCGAAGTTGAGTTG
	D. nerii	PCR	External forward	*HUV*_F06	22	CACGTAACTTGCAGAACTATCC
		PCR	External reverse	*HUV*_R05	21	CCATCATTAAAGCCGTAGTCG
	M. pyrrhosticta	PCR	External forward	Same with *HUV*_F05		
		PCR	External reverse	*HUV*_R06	22	CATCAGTAAAGCGTTGGTTGTC
	M. bombylans	PCR	External forward	Same with *HUV*_F06		
		PCR	External reverse	*HUV*_R07	24	GTATCGGTTGTCCATCAGTATAGC
	A. rubiginosa	PCR	External forward	*HUV*_F07	25	CTCACGTAACCTGCAAAACTATCCG
		PCR	External reverse	*HUV*_R08	24	CATCAAAAGTCCATCAGTAAAGCC
	T. japonica	PCR	External forward	Same with *HUV*_F05		
		PCR	External reverse	Same with *HUV*_R08		
	All	Sequencing	Internal forward	*HUV*_seqF01	19	ATATGTTCCTGAGGGCTAC
		Sequencing	Internal forward	*HUV*_seqF02	20	GAAATAAGAATAGCCAAAGC
		Sequencing	Internall reverse	*HUV*_seqR01	18	CGCTTCATGGGCAAACAC
		Sequencing	Internall reverse	*HUV*_seqR02	21	AAGCGATGCAAGCGTTAGTCA
SW	*M. gaschkewitschii*	PCR	External forward	*HSW*_F01	24	CTGTAAAACATAACACCTCCGTCG
		PCR	External reverse	*HSW*_R01	24	GTACTATGGCAACTCATTAAAGCC
	A. ochracea	PCR	External forward	*HSW*_F02	24	GCTGTAGCAAATAACTTTCCTTCG
		PCR	External reverse	*HSW*_R02	24	AAATATCAACCAGAACCCAATCCC
	C. hylas	PCR	External forward	*HSW*_F03	25	AGCGACTAGTAGTCGGACTACTCAC
		PCR	External reverse	*HSW*_R03	24	GCTTGTGAGACTATGATGATTTCC
	H. affinis	PCR	External forward	*HSW*_F04	23	TTAGTCGGACTACTCACTTCGTC
		PCR	External reverse	*HSW*_R04	24	CGCTTCAAGTTCTTTGAATATCCC
	N. himachala	PCR	External forward	*HSW*_F05	24	CAAGTGAGAAGCGACTTGTAGTCG
		PCR	External reverse	*HSW*_R05	24	TATTTCCTAAGAATGCGCGTCATG
	D. nerii	PCR	External forward	Same with *HSW*_F03		
		PCR	External reverse	*HSW*_R06	25	ACAACATCAAATGTCGCTCTATCTG
	M. pyrrhosticta	PCR	External forward	Same with *HSW*_F04		
		PCR	External reverse	*HSW*_R07	24	AAGGTAATTTTGGTATGGTGGGTG
	M. bombylans	PCR	External forward	Same with *HSW*_F03		
		PCR	External reverse	*HSW*_R08	24	CTTTTGTCTTCATCTCGTTACACG
	A. rubiginosa	PCR	External forward	*HSW*_F06	25	TCTGCGACTTGTAGTCGGACTACTC
		PCR	External reverse	*HSW*_R09	25	CTCCAATGACTTTGTAGCTGGTATG
	T. japonica	PCR	External forward	Same with *HSW*_F03		
		PCR	External reverse	*HSW*_R10	24	TGTGGGTAGAAGAGAATTGATTGG
	All	Sequencing	Internal forward	*HSW*_seqF01	18	GGGTTTCCTGACGACGTG
		Sequencing	Internal forward	*HSW*_seqF02	19	AGCGTTGAGATCAGGATAG
		Sequencing	Internall reverse	*HSW*_seqR01	20	ATCTTCTTGGCTTGCTCTTG
		Sequencing	Internall reverse	*HSW*_seqR02	19	GCCAGGTTTATCACGAACA
LW	*M. gaschkewitschii*	PCR	External forward	*HLW*_F01	25	GTAATAACCATCTCCAAGCGACTTC
		PCR	External reverse	*HLW*_R01	24	TAATAAAGAACATCGCTTCGGCAC
	A. ochracea	PCR	External forward	*HLW*_F02	23	CATCTCCAAGCGATTTCCCCTAC
		PCR	External reverse	*HLW*_R02	24	TAATAAAGAACATCGCTTCGGCAG
	C. hylas	PCR	External forward	*HLW*_F03	24	GTAATAACCATCTCAAAGCGACTC
		PCR	External reverse	Same with *HLW*_R02		
	H. affinis	PCR	External forward	Same with *HLW*_F03		
		PCR	External reverse	Same with *HLW*_R02		
	N. himachala	PCR	External forward	*HLW*_F04	24	CTACAGGAATTGTGTTGATACTCC
		PCR	External reverse	*HLW*_R03	24	GAACATCGCTTCGGCAAAATCGTG
	D. nerii	PCR	External forward	*HLW*_F05	22	TCTCCAAGCGACTCCCTTATCC
		PCR	External reverse	Same with *HLW*_R02		
	M. pyrrhosticta	PCR	External forward	*HLW*_F06	24	CATCTCTAAGCGATCCCTATCCTG
		PCR	External reverse	*HLW*_R04	24	TAATAAAGATCATCGCTTCGGCAG
	M. bombylans	PCR	External forward	Same with *HLW*_F06		
		PCR	External reverse	Same with *HLW*_R02		
	A. rubiginosa	PCR	External forward	Same with *HLW*_ F05		
		PCR	External reverse	Same with *HLW*_R02		
	T. japonica	PCR	External forward	Same with *HLW*_ F05		
		PCR	External reverse	Same with *HLW*_R02		
	All	Sequencing	Internal forward	*HLW*_seqF01	19	TGCCCGAAGGAAACATGAC
		Sequencing	Internal forward	*HLW*_seqF02	21	ACCATTTCCTTGTGGTTCATG
		Sequencing	Internall reverse	*HLW*_seqR01	20	TCATTTTCTTAGCCTGTTCC
		Sequencing	Internall reverse	*HLW*_seqR02	20	TAACAACCATAGCTGGAGAC

Journal of Experimental Biology • Supplementary information

Table S3. Parameters of the absorption spectra of visual and screening pigments in the hawkmoth species

		Visual pigments									Screening pigment					
Model	Species	UV λ_{max} (nm)	Mean ($\Delta\lambda_{max}$ from Noct.) (nm)	SW λ_{max} (nm)	Mean ($\Delta\lambda_{max}$ from Noct.) (nm)	LW λ_{max} (nm)	Mean ($\Delta\lambda_{max}$ from Noct.) (nm)	UV amplitude f_{UV} (relative ratio, %)	SW amplitude f_{SW} (relative ratio, %)	LW amplitude f_{LW} (relative ratio, %)	μ_1 (nm)	σ_1 (nm)	p_1	μ_2 (nm)	σ_2 (nm)	p_2
$S_a(\lambda) = S(\lambda) - A(\lambda)$	*M. gaschkewitschi* (n = 20)	353.1	350.6	448.3	448.4	534.4	534.9	0.633 (24.4%)	0.606 (23.3%)	1.358 (52.3%)	459.8	154.5	0.634	652.3	63.9	-0.269
	T. japonica (n = 19)	348.1		448.4		535.4		0.626 (28.2%)	0.434 (19.5%)	1.162 (52.3%)	0.0095	543.2	0.344	689.1	63.8	-0.168
	C. hylas (n = 18)	344.5	346.7 (-3.9)	431.3	439.4 (-9.0)	513.0	520.3 (-14.6)	0.837 (32.5%)	0.575 (22.3%)	1.163 (45.2%)	408.8	107.2	0.771	533.1	73.1	-0.262
	M. pyrrhosticta (n = 18)	348.8		447.5		527.5		0.511 (22.5%)	0.539 (23.8%)	1.218 (53.7%)	390.7	125.9	0.429	653.0	34.6	-0.048

Journal of Experimental Biology • Supplementary information
